# Comparing Repeated (Annual) Couples HIV Testing and Counseling to Individual HIV Testing and Counseling Among Male Couples at High Risk of HIV Infection: Protocol for a Randomized Control Trial

**DOI:** 10.2196/53023

**Published:** 2024-02-13

**Authors:** Tyrel J Starks, Kory Kyre, Juan Castiblanco, Jayelin N Parker, Erin Kahle, Rob Stephenson, Demetria Cain

**Affiliations:** 1 Department of Psychology Hunter College City University of New York New York, NY United States; 2 Doctoral Program in Health Psychology and Clinical Science Graduate Center of CUNY New York, NY United States; 3 Department of Obstetrics and Gynecology University of Michigan Medical School Ann Arbor, MI United States; 4 Department of Health Behavior and Biological Sciences School of Nursing University of Michigan Ann Arbor, MI United States; 5 The Center for Sexuality and Health Disparities School of Nursing University of Michigan Ann Arbor, MI United States

**Keywords:** club drugs, counseling, dyadic interventions, emerging adults, gay and bisexual men, HIV, male couples, men who have sex with men, randomized controlled trial, RCT, relationships

## Abstract

**Background:**

Couples HIV testing and counseling (CHTC) is now a standard of care prevention strategy recommended by the Centers for Disease Control and Prevention for sexual minority men (SMM) in relationships. Despite standard recommendations that couples complete CHTC every 6-12 months, no study has empirically evaluated the effects associated with CHTC retesting.

**Objective:**

This study aims to understand the benefits associated with continued dyadic engagement in the HIV prevention continuum through routine CHTC retesting, which is of particular importance for emerging-adult SMM in relationships who use drugs.

**Methods:**

Eligible couples for this CHTC retesting trial must already be enrolled in the 4Us trial, where they completed a CHTC session after their baseline survey. The purpose of the original 4Us trial was to test the efficacy of 2 intervention components for CHTC: a communication skills training video and a substance use module. Couples were eligible for the original 4Us trial if they identified as cisgender male, were in a relationship for 3 months or longer, were aged 17 years or older, and communicated in English. At least 1 partner had to be aged 17-29 years, report HIV negative or unknown serostatus, report use of at least 1 drug (cannabis, cocaine or crack, crystal methamphetamine, ketamine, gamma-hydroxybuterate [GHB], psychedelics, ecstasy, prescription medication misuse, opiates, and nitrates) use, and engage in condomless anal sex (CAS) acts with a casual partner or have a main partner who is nonmonogamous or serodiscordant. Those who complete the 4Us 12-month follow-up and remain in a relationship with the partner they participated in 4Us with are offered the opportunity to participate in this CHTC retesting trial. Those consenting are randomized to either CHTC retesting or individual HIV testing. Follow-up assessments are conducted 3 and 6 months after randomization to evaluate the effects of repeat CHTC on 2 primary outcomes: (1) CAS with a casual partner in the absence of preexposure prophylaxis (PrEP), and (2) CAS with a serodiscordant main partner who is not virally suppressed or concurrent CAS between main and casual partners in the absence of PrEP.

**Results:**

The CHTC retesting trial launched in January 2023, and enrollment is ongoing. As of February 2024, the study had enrolled 106 eligible participants (n=53 couples).

**Conclusions:**

Findings from this CHTC retesting study will contribute to knowledge about the benefits associated with regular (repeated) CHTC testing versus routine individual HIV testing for SMM in relationships. The results of this trial will inform CHTC retesting guidance.

**Trial Registration:**

ClinicalTrials.gov NCT05833074; htps://www.clinicaltrials.gov/study/NCT05833074

**International Registered Report Identifier (IRRID):**

DERR1-10.2196/53023

## Introduction

### Background

Sexual minority men (SMM), a group that encompasses gay, bisexual, and other men who have sex with men, account for 69% of new HIV infections in the United States [1]. Despite the emergence of novel biomedical prevention options (eg, preexposure prophylaxis [PrEP], postexposure prophylaxis [PEP], and HIV undetectable=untransmittable, or treatment as prevention), this overall rate has changed little over the past decade [2]. The majority (68%) of these new infections occur among SMM under the age of 35 years [2]—a proportion that was largely unchanged in the decade between 2009 and 2019 [2].

Nearly 2 decades of research have indicated that HIV prevention interventions that address the needs of SMM in relationships are an essential component of any comprehensive US national HIV prevention plan. Findings first emerged in the early 2000s, indicating that primary partnerships were often a risk for HIV infection [3-5]. Subsequently, epidemiological modeling estimated that between 35% and 68% of HIV infections among SMM are among main partners [6-8]. Rates of primary partner transmission were particularly high (accounting for between 79% and 84% of new infections) among emerging adult SMM (aged 18-29 years) [6]. More recently, Starks et al [9] found that SMM in nonmonogamous relationships (where sex with outside partners is permitted in some way) engage in condomless anal sex (CAS) with casual partners at rates comparable to those who are single. Although men in monogamous relationships were less likely to engage in CAS with casual partners, those who do have CAS with casual partners report doing so more frequently than nonmonogamous men [9].

As evidence of the risk for HIV acquisition in couples has increased, couples HIV testing and counseling (CHTC) has emerged as a standard of care prevention strategy recommended by the World Health Organization and is now considered a proven and effective public health strategy by the US Centers for Disease Control and Prevention [10]. Protocols have been adapted for SMM in the United States [11] and demonstrated safety (with no evidence of increasing intimate partner violence [IPV] among those participating in CHTC) [12]. During CHTC, couples receive all elements of the counseling, testing, and results delivery together. In addition, the HIV tester facilitates a future-oriented dialogue between the partners about their HIV prevention practices, sexual agreement, and communication related to HIV risk. The goal is for the couple to leave with a shared vision—a set of agreed-upon actions—about HIV prevention and relevant sexual practices [13,14]. CHTC has shown marginally significant reductions in HIV-related sexual risk-taking in men who test HIV negative [15] as well as men who were newly diagnosed with HIV [12,15]. A recent trial showed that participation in CHTC also increased rates of viral suppression among serodiscordant male couples [16].

There are 3 rationales that indicate the potential importance of CHTC retesting. First, sexual health outcomes for relationship partners are interdependent. A coordinated effort maximizes prevention outcomes for both partners. Second, the potential for fluidity and change in behavior means that ongoing communication about sexual behavior and dyadic participation in HIV prevention is essential for partners to maintain effective coordination. Third, CHTC works by activating prevention communication among partners, conferring benefits for SMM in relationships that routine individual HIV testing cannot.

### The Interdependence of Sexual Health Outcomes

Much of the research on couples HIV prevention has been organized by interdependence theory [17] and dyadic coping [18]. Within this framework, HIV prevention can be understood as a shared or joint goal, one that requires effort from both partners to be accomplished. The shared nature of this goal is evident in data on main partner HIV transmission risk behavior (TRB). If 1 partner in the relationship engages in sexual risk behavior leading to HIV infection, or if 1 partner in the relationship is not aware that he is living with HIV for whatever reason, the likelihood of transmission between main partners is high. In short, prevention outcomes for both partners in a relationship are maximized when they coordinate or contribute joint effort to accomplish HIV prevention goals.

Interdependence theory [19] suggests coordination is easier for couples with better relationship functioning and communication. Interdependence theory suggests that in relationships characterized by a high degree of satisfaction, commitment, and emotional investment, partners are more likely to consider the impact of their behavior on one another and their relationship overall. This transformation of motivation, away from a focus on personal priorities and toward a consideration of one’s partner, enhances partners’ motivation to reach consensus around shared sexual agreements and related HIV prevention plans. It also provides the impetus for partners to then support one another in adhering to these agreements or accomplishing the goals implied within them.

### The Importance of Ongoing Communication—Why One-Time Agreement on a Goal Is Not Enough

Research on the prevalence of sexual agreements in male couples suggests a substantial number disagree about the nature of their sexual agreement. For example, 1 partner believes that sex with outside partners is in some way permitted; meanwhile, the other believes they are monogamous. While estimates vary widely, Stephenson and colleagues [20] found that, even in a sample of couples where 84.7% of men reported having an agreement, partners had discrepant perceptions of what that agreement was in 58.7% of couples. Perhaps not surprisingly, couples with discrepant perceptions of their sexual agreement score lower on measures of adaptive communication compared to those whose perceptions are aligned. The completion of CHTC resolves discrepant perceptions by catalyzing direct, explicit communication about the couple’s rules and understandings related to sex with outside partners.

Once formed, sexual agreements and HIV prevention plans are neither fixed nor static. They have the potential to change over time as the needs and priorities of the individual partners in the couple evolve. Stephenson and colleagues [21] recently found that just 6 months after completing CHTC, partners in 22.6% of couples had discrepant perceptions of their sexual agreements, and 12.7% had broken (or failed to fully adhere to) their agreement. Cross-sectional studies consistently indicate that couples who have been together longer are more likely to develop sexual agreements that permit sex with outside partners [9,22-24]. This trend has been observed even in samples of couples that are age-restricted to emerging adulthood [25]. Some findings also show that partners reporting discrepant perceptions of their sexual agreement and breaking their agreement increase with relationship length [24].

This potential for change over time necessitates ongoing communication about sexual agreements and HIV prevention practices to maintain partners’ alignment and coordination and underscores the need for repeat CHTC. Unfortunately, several factors complicate HIV prevention communication for relationship partners. In general, partnered SMM perceive themselves to be at lower risk of HIV infection and test for HIV less often compared to single SMM [26,27]. For at least some couples, forfeiting HIV prevention, engaging in CAS together, or stopping PrEP is interpreted as an indicator of commitment or emotional closeness [28-31]. As a result, the introduction of condom use, PrEP, or PEP to prevent HIV infection is complicated by the potential that it might convey a lack of commitment to or trust in their partner [32-37] for these couples.

### For SMM in Relationships, CHTC Retesting May Confer Specific Benefits Above and Beyond Those Associated With Routine Individual HIV Retesting

During CHTC, partners obtain updated information on their own and one another’s HIV status. As part of routine CHTC retesting, the CHTC provider can reinforce the couple’s relationship functioning. The CHTC provider also initiates a conversation among partners with the goal of updating their HIV prevention plan and clarifying any changes in behavior or perception that would impact the couple’s sexual agreement. These provider-initiated interactions may be of critical importance in couples where 1 partner desires to discuss such changes but is concerned that raising the topic might harm the relationship or is uncertain about how to initiate conversation.

Understanding the benefits associated with continued dyadic engagement in the HIV prevention continuum through routine CHTC retesting is of particular importance for emerging adult SMM in relationships who use drugs. Rates of illicit drug use are generally higher among SMM (11.1% to 27.1%) compared to heterosexual men (5.7% to 16.2%) [38,39]. Rates of cannabis use are also higher among SMM (36%) compared to heterosexual men (20.4% to 24.7%).

High rates of drug use and associated sexual risk-taking extend to partnered SMM, particularly those in nonmonogamous agreements. SMM in nonmonogamous relationships consistently report rates of illicit drug use that are comparable to single SMM; meanwhile, men in monogamous relationships are significantly less likely to use illicit drugs [9,22,23,40]. Extensive research has shown that SMM who use cannabis and illicit drugs are more likely to have CAS [41-44] with casual partners. Associations between drug use and sexual risk-taking are comparable for partnered and single SMM. Our research suggests the association between illicit drug use (excluding cannabis) and the odds of CAS with casual partners is comparable for single SMM and those in nonmonogamous agreements [9]. Meanwhile, the association between cannabis use and the odds of CAS with casual partners is comparable for single SMM and those in monogamous agreements—for whom the behavior breaks their agreement. This converges with other evidence suggesting that drug use during sex is associated with breaking a sexual agreement [45] and decreased condom use among partnered SMM [40,46].

### Objective

The purpose of this study is to evaluate the efficacy of annual CHTC retesting to reduce indicators of sexual risk relative to routine individual HIV testing and counseling among male couples.

## Methods

### Trial Design

This study uses a randomized controlled trial design integrated with the ongoing 4Us trial [47]. The purpose of the original 4Us trial is to test the efficacy of 2 intervention components for CHTC: a communication skills training video and a substance use module. Couples are eligible for the original 4Us trial if at least 1 partner reports HIV negative or unknown serostatus, reports at least 1 drug, and engages in HIV TRB. Participants in this ongoing trial (4Us) complete an individual baseline assessment, after which couples complete a CHTC session following the standard CHTC protocol. In a full-factorial design, half of the couples are randomly assigned to complete an adjunct module addressing drug use, and half are assigned to view an assertive communication training (ACT) video (later referred to respectively as the substance use calendar and ACT video). This results in 4 study conditions (CHTC as usual, CHTC plus the substance use calendar, CHTC plus the ACT video, and CHTC plus both adjunct components). Follow-up assessments occur 3, 6, 9, and 12 months post intervention using procedures analogous to the baseline. Participants who complete the 12-month follow-up of the 4Us trial and meet eligibility criteria for the CHTC retesting trial are consented individually to participate in this protocol. If both couples consent into this protocol during their 12-month assessment, they are randomly assigned to either couples retesting or individual HIV testing. Those randomized to couples retesting complete the same 4Us condition they were assigned at baseline. Follow-ups are then completed 3 and 6 months later (15 and 18 months post completion of the baseline in the original 4Us trial).

### Rationale for Comparison Condition (Individual HIV Testing and Counseling)

Current Centers for Disease Control and Prevention (CDC) recommendations advise that all individuals between the ages of 13 and 64 years should be tested for HIV at least once. Individuals with additional risk factors, including men who have sex with men, are advised to test at least annually [48]. Recommendations for retesting every 3-6 months apply to the highest-risk groups. CDC has summarized the benefits of routine individual HIV testing [48]. Routine testing reduces the onward transmission of HIV infection. It is estimated that 40% of new HIV infections are transmitted by individuals who do not know they are HIV positive. HIV diagnosis is a prerequisite to the initiation of antiretroviral treatment (ART) for those who are living with HIV, and achieving viral suppression through ART nearly eliminates the likelihood of sexual HIV transmission. For those who learn they are HIV-negative, routine HIV testing presents an opportunity to discuss HIV prevention options and risk reduction practices.

### Study Setting

All research staff are based at a university research center at Hunter College of the City, University of New York. All study assessments and intervention sessions are conducted remotely through Zoom (Zoom Video Communications, Inc), a Health Insurance Portability and Accountability Act (HIPAA)–compliant videoconferencing software.

### Eligibility Criteria

All participants must be enrolled in the original 4Us trial [47] and complete their assigned intervention condition after baseline. Eligibility criteria for the original 4Us trial necessitate that partners in a couple identify one another as “main partners.” In addition, both partners in each couple must identify as cisgender male, be 17 years of age or older, and have a US residence. In addition, at least 1 participant in each couple must (1) be aged 17-29 years; (2) self-report HIV-negative or unknown serostatus; (3) report use of at least 1 illicit drug (cocaine or crack, opiates, misuse of prescription medication, stimulants, psychedelics, ecstasy, ketamine, and GHB) in the past 30 days; (4) have engaged in CAS with a casual partner or a main partner who is nonmonogamous or serodiscordant in the past 90 days; and (5) be able to speak and read in English. If a serodiscordant couple enrolls in the study, the partner living with HIV is not asked to submit viral load test results.

To be eligible for participation in this CHTC retesting study, participants must complete the 12-month follow-up assessment associated with the 4Us trial [47]. In addition, they must remain in a relationship with the same partner with whom they completed their intervention session after baseline in that trial, and that partner must also agree to enroll in this study.

Participants will be excluded from the study if they indicate that they are single or if they are in a relationship with a new main partner (different from the person with whom they completed their initial 4Us intervention session with the following baseline). Participants will also be excluded if they exhibit signs of serious mental illness or cognitive deficit that impair their functioning during routine research interactions; report IPV with their main partner accompanied by ongoing safety concerns in the current relationship; or indicate being coerced to participate.

### Recruitment and Enrollment

Following completion of their 12-month follow-up in the 4Us trial, [47] eligible participants will be offered the opportunity to enroll in this study. Those who are interested will be given a separate consent form detailing the purpose and activities involved. Assessments are conducted separately with each partner in a couple. The first partner in a couple to complete their 12-month follow-up and consent to continue in this study will be told that the couple’s intervention session will be scheduled after their partner also completes the follow-up and pending their partner’s individual consent to participate.

### Interventions

All participants will be randomly assigned to either CHTC retesting or individual rapid HIV testing session. Intervention sessions for both conditions are conducted remotely over Zoom. All sessions are audio-recorded for data collection, fidelity monitoring, and supervision.

#### CHTC Retesting

CHTC retesting involves the completion of the CDC standard CHTC protocol [49]. This includes (1) introducing the CHTC process and receiving testing consent from the couple, (2) explaining the HIV test and potential results and collecting the sample, (3) building rapport by exploring the couple’s relationship, (4) discussing HIV risk concerns and reasons for testing, (5) discussing the couple’s sexual agreement and how they handle sex outside of the relationship, (6) providing the results to each partner in the couple, (7) developing a care, treatment, and prevention plan based on results, and (8) providing referrals as needed. Each participant provides a saliva sample for an Oraquick Home testing kit. The test develops during the CHTC session while the counselor discusses steps 3 through 5.

Participants assigned in the original 4Us trial to a condition that includes viewing ACT videos before completing CHTC. The ACT video portrays 4 couples in scenes discussing HIV testing, drug use, sexual agreements, and drug use during sex. Each scene is depicted twice. The initial viewing shows the couple making one or more communication errors. The second viewing shows the couple using more effective communication skills, resolving the situations more adaptively.

Participants assigned to a condition that includes the substance use calendar module complete this module just before the delivery of HIV testing results. The substance use calendar occurs in the CHTC session before the HIV test results are given. The couple is asked to collectively complete a 30-day calendar of daily drug and alcohol use. The counselor provides the calendar on a shared computer screen as a Microsoft Excel (Microsoft Corporation) document. After the completion of the calendar, the counselor engages the couple in a discussion about their use, establishes the couple’s goals and limits for drug use, and makes plans to achieve these goals. Additional details about the content of these intervention components are available elsewhere [47]. The intervention session in the CHTC retesting study follows the same procedures as the original 4Us trial. The same counselor may or may not conduct the resting intervention, which mirrors how testing may occur in a community setting.

#### Individual Rapid HIV Testing Session

Individual rapid HIV testing session, also known as the individual HIV counseling, testing, and referral (CTR), is the current standard of care for individual testing and first involves preparing to conduct an HIV test (explaining the HIV test and possible results the participant could receive). After answering any participant questions and gaining consent to conduct the test, the HIV counselor walks the participant through the rapid, oral HIV test. While the HIV test is processing for 20 minutes, the counselor and participant discuss a prevention plan, avoiding the topic of their partner and steering the conversation to address only the individual. HIV-negative individuals are given a standard referral list for future HIV testing, sexually transmitted infection (STI) testing, and PrEP options. Those testing HIV positive are counseled about their test result and referred to confirmatory testing. When serodiscordant couples are randomized to individual rapid HIV testing control condition, individuals who are living with HIV do not receive HIV testing. Instead, they receive information about ART adherence, undetectable=untransmittable, and STI testing.

### Intervention Training

As part of the ongoing 4Us trial [47], all interventionists complete Sullivan et al [11] CHTC training curriculum (adapted by the CDC) as well as training in motivational interviewing. Each counselor role played motivational interviewing–spirited CHTC at least 3 times with fellow project staff and at least once with principal investigators TJS and RS. All role-play practice sessions were audio-recorded for training purposes. Booster training was offered at least once per year for counselors by TJS. In addition to CHTC and motivational interviewing training, counselors were refreshed on individual HIV CTR during 2 half-day training sessions. During these trainings, counselors practiced through a plethora of role-play scenarios, and a standard protocol was developed. These trainings were followed by at least 3 audio-recorded, role-play practice sessions with fellow project staff. TJS conducted individual and group feedback and skills coaching sessions for the CTR counselors. Following training, the counselors maintained weekly supervision with TJS.

### Fidelity Monitoring and Supervision

TJS provides weekly supervision to study counselors. These sessions involve a review of participant CHTC session audio recordings, discussion, and additional skills training as needed. In addition, 20% of CHTC session recordings are assessed using the CDC’s CHTC fidelity checklist [11]. Separately, fidelity to motivational interviewing during delivery of the substance use calendar is assessed using the Motivational Interviewing Treatment Integrity system [50] with supplemental codes developed by Starks et al [51].

### Primary Outcomes

Primary analyses focus on 2 (individual-level) behavioral indicators of HIV transmission risk: (1) the number of CAS acts with a casual partner in the absence of the respondent taking PrEP and (2) CAS with a serodiscordant main partner who is not virally suppressed or concurrent CAS between main and casual partners in the absence of the respondent taking PrEP. The availability of day-level data generated by the timeline followback (TLFB) assessment allows for an examination of event-driven PrEP dosing as well as overall day-level adherence. Self-reported sexual behavior will be corroborated with results from bacterial STI (gonorrhea and chlamydia) testing. In instances where these indicators of HIV TRB suggest CAS with a casual (or main) partner occurred but was not reported, we will use data from the objective indicator.

### Secondary Outcomes

Secondary analyses focus on indicators of drug use and severity (primary outcomes in the parent study). Drug use is operationalized in 2 ways. First, using TLFB interview data, the quantity of drug use will be operationalized using the total number of instances reported during the assessment period. We will examine cannabis and other illicit drug use frequency separately. Urine assay will corroborate the self-report. In instances where urine results signal the use of drugs not reported, we will use data from the objective indicator. Second, drug use severity will be assessed using the Drug Abuse Screening Test [52] total score.

### Participant Timeline

**Figure 1:**
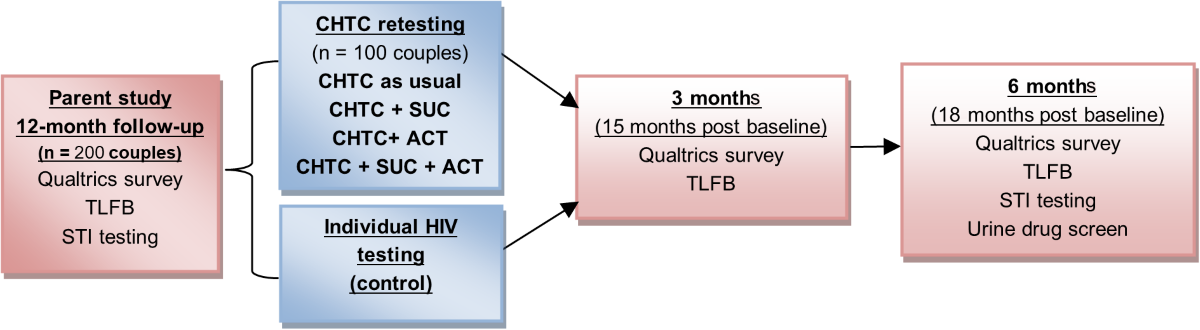
Timeline of participation. ACT: assertive communication training; CHTC: couples HIV testing and counseling; STI: sexually transmitted infection; SUC: substance use calendar; TLFB: timeline followback.

See Figure 1 for a timeline of participant flow through the study.

### Sample Size

Power to detect significant between-group differences in primary outcomes was calculated using the PASS program (version 2022; NCSS, LLC). Analyses focused on power to detect a significant between-group difference at any 1 follow-up time point. Results from the tests for difference between 2 Poisson rates in a cluster randomized design module indicated the study had power of >0.80 to detect a 20% or greater reduction in the rate of CAS with casual partners. This estimate assumed a sample of 200 couples with equal allocation to condition, and models were tested with based rates of 2-4 instances of CAS with a casual partner among the control (individual HIV testing and counseling) condition. Results from the tests for 2 proportions in a cluster-randomized design suggested proposed study has power of >0.80 to detect a 22% reduction in the proportion of reporting main partner TRB, assuming a sample of 200 couples and a 70% base rate in the control condition.

### Assignment of Interventions

#### Randomization

Couples will be randomly assigned to 1 of 2 conditions, CHTC retesting or individual rapid HIV testing. Randomization will be stratified for previous randomization in the 4Us trial (CHTC as usual, CHTC plus the substance use calendar, CHTC plus the ACT video, and CHTC plus both adjunct components). The random assignment will be performed by the project manager, who will enter the stratification criteria in Qualtrics to obtain the condition before intervention delivery.

#### Blinding

Intervention staff cannot be blinded to the condition they are delivering. Likewise, participants cannot be blinded to the condition. The 12-month follow-up in the 4Us trial (which serves as the preintervention data point for this study) is completed before participants are consented to this study. Assessment staff are blinded to condition at follow-up. Where blinding is possible, unblinding is not anticipated.

### Data Collection, Management, and Analyses

#### Data Collection for Primary Outcomes

##### Self-Reported Sexual Behavior

Procedures are the same as those used in the ongoing 4Us protocol [47]. Research assistants gather self-reported sexual behavior data for the past 30 days using a structured TLFB interview (Sobell and Sobell [53]). A Microsoft Access (Microsoft Corporation) database is used to facilitate the capture of data, including “anchor dates” or significant events; missed PrEP doses (for those on PrEP); heavy drinking and drug use; and sexual events. Sexual event data further comprise partner type (main or casual), the sex act performed (eg, anal insertive and anal receptive), and whether a condom was used.

##### Biological Testing

The Molecular Testing Laboratory (MTL) coordinates at-home STI testing. Materials necessary for collection and return shipping are delivered before the scheduled assessment meeting with the research assistant. Specimen collection is completed at a time designated in the assessment. The research assistant is available to review collection procedures, confirm the accuracy of shipping information, and observe the packaging of specimens for shipment.

The presence of urethral STIs will be tested in urine specimens; meanwhile, the presence of rectal STIs will be tested in (self-administered) rectal swab. The Abbott RealTime CT/NG assay is used to evaluate the presence of *Chlamydia trachomatis* and *Neisseria gonorrhoeae*. This is a Food and Drug Administration (FDA)–cleared real-time polymerase chain reaction assay for direct, qualitative detection of a region of the cryptic plasmid DNA of *C trachomatis* and the Opa gene of *N gonorrhoeae*.

##### HIV Testing

HIV testing, during CHTC and individual HIV testing sessions, will be performed using the Oraquick 4th generation testing kit. These kits are approved for at-home self-testing, and FDA-approved instructions are included. At follow-up (6 months), HIV testing will be conducted individually using self-administered dried bloodspot, giving participants the option of an Oraquick HIV to complete their HIV test if they prefer.

##### Retention Plan

Retention procedures continue those used in the ongoing 4Us trial [47], from which participants for this study are recruited. Assessments are conducted individually to retain participants who break up and whose relationship may have ended during the course of their participation in the study and to reduce the burden associated with coordinating a single assessment session with both partners. Study staff have at least quarterly contact with participants through email, SMS text messaging, and telephone (based on participant preference), which serves to maintain engagement and the accuracy of contact information over the study period.

#### Data Management

TLFB data are gathered by a trained interviewer using a data-entry system programmed in Microsoft Access. The institutional review board of Hunter College has reviewed all study procedures. In addition, procedures are reviewed by the Data Safety Monitoring Board (DSMB), which consists of experts in sexual health and substance use intervention research with SMM.

#### Data Analysis Plan

##### Data Screening Procedures and Analyses of Attrition

We will follow standard procedures for cleaning data and assess whether variables conform to the distributional assumptions of our analyses. The 12-month follow-up assessment in the parent study serves as prerandomization (ie, baseline) data for the purposes of these analyses. We will begin with a sensitivity analysis to examine whether couples enrolled into the randomized controlled trial proposed in this revision differ from those participants in the parent study (DA050508) who are not enrolled either due to relationship termination or lack of interest. This analysis will serve to identify behavioral factors (eg, drug use or sexual risk-taking) and relationship factors (eg, satisfaction, commitment, or communication skills) that are associated with relationship termination and uptake of CHTC retesting (among those couples who remain together). Subsequently, we will conduct an analysis of randomization success and attrition to determine if either is associated with (1) demographic variables or (2) drug use or TRB outcomes assessed at the 12-month 4Us follow-up. Factors that are observed to covary significantly with randomization or attrition will be incorporated in outcome analyses.

##### Primary Analyses of Intervention Effects

The primary hypothesis is that CHTC retesting will be associated with significant reductions in sexual HIV TRB (primary) outcomes compared to individual HIV testing. Using procedures similar to our previously published studies [54], outcome analyses will be conducted in a multilevel modeling framework. Analyses will use full-information maximum likelihood estimation and specify a negative binomial distribution consistent with the count nature of primary outcomes. The appropriateness of a Poisson distribution will be determined by inspection of the significance of the dispersion parameter in negative binomial models. This accounts for the nesting of individuals within couple. Because randomization occurs at the couple level, condition is a level 2 variable. The effect of the intervention will be evaluated by examining the regression coefficient (and associated *P* value). Separate models will be calculated to predict outcomes at 3- and 6-month follow-ups (with Bonferroni correction for repeated nonorthogonal tests). Mplus (Muthén and Muthén) accommodates count and dichotomous outcomes within the multilevel modeling context. Analysis of secondary outcomes will follow analogous analytic procedures.

##### Moderation or Mediation Analyses

Where possible, we will evaluate whether retesting using the adjunct (substance use calendar and ACT video) components is associated with specific reductions in primary (HIV TRB) outcomes. While the primary goal of this analysis is to test whether CHTC retesting, in general, is associated with benefits above and beyond individual HIV testing, the study design permits a preliminary examination of whether retesting that involves these adjunct components developed by our team is associated with additional benefits above and beyond retesting using the standard CHTC protocol. We will conduct a preliminary examination of this possibility using analytic procedures analogous to those used to evaluate effects on secondary drug use outcomes.

##### Data Monitoring

The DSMB, comprising 3 independent experts in the fields of HIV prevention, substance use intervention, and biostatistics, was convened in accordance with NIH policy to oversee the original 4Us trial (NCT05000866). The DSMB convenes at least annually in response to the occurrence of any serious adverse events.

Study staff will monitor the occurrence of 2 anticipated adverse events, including HIV incidence and IPV. Participants provide data on these at each follow-up, and responses are reported to the DSMB annually by the primary investigator. The primary investigator will report unanticipated events and those reported spontaneously by participants to the DSMB on an ongoing basis. These are also summarized in their annual meeting report.

##### Trial Modification and Discontinuation

There are no plans to conduct interim analyses, and no a priori stopping rule has been established for this trial. Randomization may be discontinued, and the trial stopped under the guidance of the DSMB in response to adverse event review. Sponsor and DSMB approval are required for substantive changes to trial design. Changes also require institutional review board approval before implementation, and the clinicaltrials.gov record would be updated to reflect modifications.

##### Confidentiality

Unique study ID numbers, assigned in the 4Us trial, are used to link participant data. Only essential study staff have access to the password-protected file that links contact information to study ID. All materials will be stored in databases that are HIPAA-compliant. Laboratory test results are ordered and received through a HIPAA-compliant platform maintained by the MTL. Participants consent to having the MTL conduct state-level, name-based reporting for positive HIV or STI results.

All study-affiliated staff complete mandatory training in good clinical practice and the responsible conduct of research. Standard operating procedures are created to minimize breaches in confidentiality. Participants indicate their preferred mode of communicating with staff (eg, telephone call, SMS text messaging, email) and may request that study staff use discretion when leaving messages. As a standard measure, NIH grants a federal certificate of confidentiality.

##### Posttrial Care

The study team has compiled an index of national resources that are made available to participants. These include search engines that identify HIV prevention and care providers in the United States. Positive HIV or STI results are delivered by study staff, and linkage to care is discussed. The MTL also complies with state-level, name-based reporting procedures, and local or state department of health staff may follow up in some instances. Participants who exhibit signs of serious mental illness or clinically significant distress in interactions with study staff will be evaluated by a study team member with training in mental health counseling and crisis risk assessment.

##### Dissemination

The authorship team is committed to the dissemination of study results. This will be accomplished through several mechanisms. First, we convey information on study activities and progress to participants through a newsletter to participants. Second, the investigative team regularly attends local, national, and international conferences to share findings with other researchers, service providers, and policymakers. Finally, the investigative team will prepare manuscripts for publication in peer-reviewed journal articles. Authorship in publications will be based upon intellectual contribution and guided by American Psychological Association guidelines for authorship. There are no plans to make participant-level data available to the public. Data will be available to other researchers upon request from the study principal investigator.

### Ethical Considerations

This study protocol was approved by the City University of New York’s Human Research Protection Program (2022-0630) and is registered with ClinicalTrials.gov (NCT NCT05833074). All participants who complete their original 4Us 12-month appointment, meet study eligibility, and agree to be enrolled in this CHTC retesting trial provide consent individually over Zoom. After reading the consent form, the research assistant obtains verbal consent to participate. Given the remote nature of study activities and the geographic diversity of the sample, a waiver of documentation of consent was obtained. All data are deidentified to protect participants’ privacy by assigning each participant a unique identifier. Data systems are established to only keep participants’ name and their unique identifier in a secured database. Participants are compensated US $20 for attending the intervention session, US $40 for completing the 3-month assessment (US $30 for survey and TLFB and US $10 for urine drug testing), and US $60 for completing the 6-month assessment (US $30 for survey and TLFB and US $30 for urine drug testing, STI, HIV testing, and PrEP adherence, if applicable). Compensation for this trial’s baseline is paid as the 12-month assessment for the original 4Us trial.

## Results

This study began recruitment in January 2023, and all participant components are projected to end in May 2025. As of December 2023, 102 individuals (51 couples) have enrolled in the CHTC retesting trial.

## Discussion

The results of this trial have the potential to inform a previously unstudied aspect of CHTC—the utility of repeated, annual completion. This trial will be the first experimental evaluation of CHTC retesting. Results therefore have the potential to substantively inform CHTC guidance.

At the same time, several limitations arise as a function of study design. Both partners in a couple must consent to participate in resting. We are therefore unable to compare CHTC to a condition in which 1 (but not both) partner receives an individual HIV test. In addition, this trial is subject to the limitations of the 4Us study from which participants are recruited. Briefly, the demands of dyadic participation may present barriers to study enrollment or engagement, particularly for couples with relatively lower relationship quality [55]. The sample will not include partnered SMM in relationships where one partner is unable or unwilling to participate. The 4Us sample is recruited through advertisements on social networking and dating applications. It, therefore, underrepresents SMM who do not engage in these digital spaces. Furthermore, all participants in the 4Us study are aged 17 years or older, and at least 1 partner must be aged 17-29 years. This limits the generalizability of findings to adolescents and older adults.

Despite these limitations, this study has the potential to enhance knowledge about the effects of CHTC on the sexual health of SMM couples. This may inform future efforts to disseminate the intervention in the United States and internationally, as well as to gender-diverse couples.

## Abbreviations

ACT: assertive communication training
CAS: condomless anal sex
CDC: Centers for Disease Control and Prevention
CHTC: couples HIV testing and counseling
CTR: counseling, testing, and referral
DSMB: Data Safety and Monitoring Board
FDA: Food and Drug Administration
GHB: gamma-hydroxybuterate
HIPAA: Health Insurance Portability and Accountability Act
IPV: intimate partner violence
MTL: Molecular Testing Laboratory
NIH: National Institutes of Health
OR: odds ratio
PEP: postexposure prophylaxis
PrEP: preexposure prophylaxis
SMM: sexual minority men
STI: sexually transmitted infection
TLFB: timeline followback
TRB: transmission risk behavior
